# Are thin flaps still a contraindication? Early outcomes of prepectoral DTI reconstruction in a large single-center cohort

**DOI:** 10.3389/fonc.2026.1779735

**Published:** 2026-03-06

**Authors:** Marzia Salgarello, Niccolò Lazzeri Domar, Ricci Luca, Giuseppe Visconti, Lorenzo Scardina, Sabatino D’Archi, Alba Di Leone, Gianluca Franceschini, Liliana Barone Adesi

**Affiliations:** Department of Woman and Child’s Health Sciences, Fondazione Policlinico Universitario A. Gemelli – IRCCS, Rome, Italy

**Keywords:** direct-to-implant, implant reconstruction, mastectomy, mastectomy flap thickness, prepectoral breast reconstruction

## Abstract

**Introduction:**

Prepectoral direct-to-implant (DTI) breast reconstruction has gained popularity due to reduced morbidity and improved aesthetic and functional outcomes. However, thin mastectomy flaps (<1 cm, R1 following the Rancati classification) are traditionally considered at higher risk of complications, often leading surgeons to prefer subpectoral or staged approaches. This study evaluates whether prepectoral DTI reconstruction can be safely performed in patients with thin mastectomy flaps, challenging the traditional view that these patients are unsuitable candidates.

**Materials and methods:**

A retrospective single-center analysis was conducted on 1239 patients undergoing immediate prepectoral DTI breast reconstruction after nipple-sparing (NSM), skin-sparing (SSM), or skin-reducing mastectomy (SRM) between August 2018 and June 2025 (1663 mastectomies). Patients with prior radiotherapy were excluded. Intraoperative flap thickness was categorized as Group 1 (5–6 mm), Group 2 (7–9 mm), and Group 3 (≥ 10 mm). The primary endpoint were early postoperative complications (<30 days), classified as major or minor.

**Results:**

Overall, 152 mastectomies (9.14%) developed early complications. Complication rates were comparable across flap-thickness groups (Group 1: 10.48%, Group 2: 9.57%, Group 3: 8.66%; p = 0.704). Rates of wound dehiscence, infection, implant extrusion, and minor complications did not significantly differ among the groups. Ischemic complications requiring revision were more frequent in thinner flaps (2.1% in Group 1 vs. 0.51% and 0.43% in Groups 2 and 3), although absolute numbers remained low. Periprosthetic seromas showed a statistically significant but clinically modest difference.

**Conclusions:**

Flap thickness alone is not an independent predictor of early morbidity in prepectoral DTI reconstruction. When intraoperative perfusion is adequate and a standardized protocol is used, even flaps <1 cm can be safely subjected to DTI prepectoral reconstruction. These findings support expanding prepectoral indications to selected patients with thin mastectomy flaps.

## Introduction

Breast cancer is the most prevalent malignancy among women worldwide (1). Despite increasing adoption of breast-conserving surgery, mastectomy remains a widely performed procedure (2, 3), particularly in patients younger than 40 years, BRCA mutation carriers, and in cases involving large tumors (≥5 cm), multifocal/multicentric disease, or an elevated risk of recurrence (4, 5). Given the psychological and physical impact of mastectomies, immediate reconstruction plays a crucial role in restoring body image and improving quality of life, without putting oncological safety at risk (6–8).

Implant-based reconstruction is the most widely used method (9, 10), with growing preference for direct-to-implant (DTI) placement in the prepectoral plane after nipple-sparing (NSM), skin-sparing (SSM), or skin-reducing mastectomy (SRM) (11–13). Compared with subpectoral techniques, prepectoral reconstruction preserves the pectoralis major, reduces pain, prevent animation deformity, and improves functional and aesthetic outcomes. Large series and meta-analyses confirm its safety, showing lower rates of reoperations, capsular contracture, and breast reconstruction failure, although rippling is more common (14, 15).

A key challenge remains mastectomy flap viability, with necrosis reported in up to 30% of cases (16–18). Studies investigating anatomical thresholds for safe flap thickness often classify flaps < 1 cm (R1 according to the Rancati classification (19)) as unsuitable for prepectoral placement. However, flap perfusion, vascular anatomy, and surgical technique may be more relevant predictors than thickness alone (20).

To improve outcomes, acellular dermal matrices (ADMs) have been used, but they increase risks of early complications such as seroma, infection, red breast syndrome, and costs (21, 22). Therefore, ADM-free strategies, such as polyurethane-coated implants, have gained interest with evidence showing comparable complication rates and superior patient-reported satisfaction (23–25).

Recent studies emphasize the importance of multidisciplinary preoperative evaluation and intraoperative perfusion assessment in expanding prepectoral indications (26). Nevertheless, patients with very thin flaps are frequently excluded from prepectoral reconstruction in current literature due to concerns about mastectomy flap ischemia and postoperative complications (27, 28).

This study evaluates outcomes of prepectoral DTI breast reconstruction in patients with mastectomy flaps <1 cm (R1), performed with PU-coated implants without ADM or synthetic meshes. By focusing on this traditionally high-risk subgroup, we aim to demonstrate that with careful patient selection and meticulous surgical technique, safe and effective reconstruction is achievable regardless of flap thickness.

## Materials and methods

A retrospective single-center study was conducted at Fondazione Policlinico Universitario A. Gemelli IRCCS in Rome, including 1239 consecutive patients who underwent immediate prepectoral DTI reconstruction following NSM, SSM, or SRM between August 2018 and June 2025. A total of 1663 mastectomies were performed, including 424 bilateral procedures. Preoperative assessments included anthropometric data (age, weight, height, BMI) and documentation of comorbidities.

No exclusion criteria were applied based on patient-related risk factors. Patients ≥ 65 years and those with diabetes, BMI ≥ 30 kg/m², mastectomy specimen weight ≥ 500 g, vascular disease, hypertension, autoimmune conditions, smoking habits, polypharmacy, or prior neoadjuvant chemotherapy were included. Exclusion criteria were limited to mastectomies with prior radiotherapy.

The study was approved by the Institutional Ethics Committee and conducted in accordance with the Declaration of Helsinki. Written informed consent for data collection and analysis was obtained from all participants.

### Endpoints

The primary endpoint was the incidence of early postoperative complications occurring within 30 days after surgery. Complications were classified as major or minor. Major complications were defined as events requiring surgical revision, systemic pharmacologic therapy, or hospital readmission. Minor complications included conservatively managed conditions not requiring reoperation or systemic therapy, such as seroma treated by aspiration, minor ischemia not requiring surgical revision, or superficial wound dehiscence treated with dressing change. The specific complications analyzed included seroma, mastectomy flap ischemia, wound dehiscence, infection, and implant exposure.

### Surgical technique

All patients were evaluated by a multidisciplinary surgical board that determined the most appropriate oncologic and reconstructive strategy, according to the ROME protocol (29).

Preoperative planning included bilateral breast measurements (sternal notch–nipple distance, nipple–inframammary fold distance, and nipple–nipple distance), assessment of ptosis based on Regnault’s classification (30), and documentation of any preexisting asymmetries. Estimated implant volume and mastectomy flap thickness, assessed by pinch test and mammography, were recorded for each patient.

**Figure 1:**
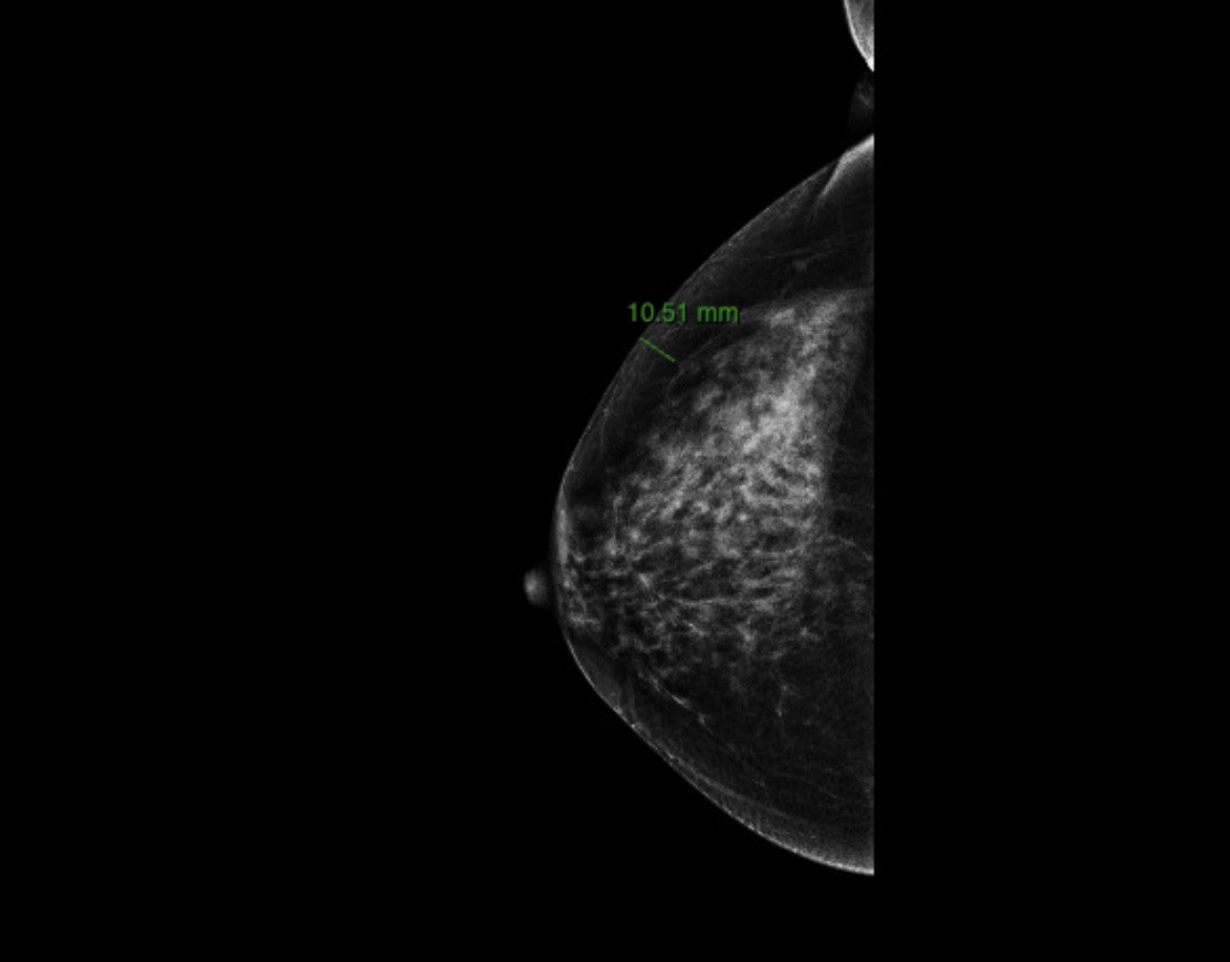
Preoperative measurement of breast tissue thickness according to the Rancati protocol. The mediolateral oblique (MLO) projection is used to assess the distance between the skin surface and the anterior lamella of the superficial fascial system across the five standardized points required to calculate the Preoperative Breast Tissue Coverage (P-BTC). These measurements form the basis for the radiological BTCC (types 1–3). An additional measurement at the level of the inframammary fold is also illustrated, providing complementary information on tissue coverage.

**Figure 2:**
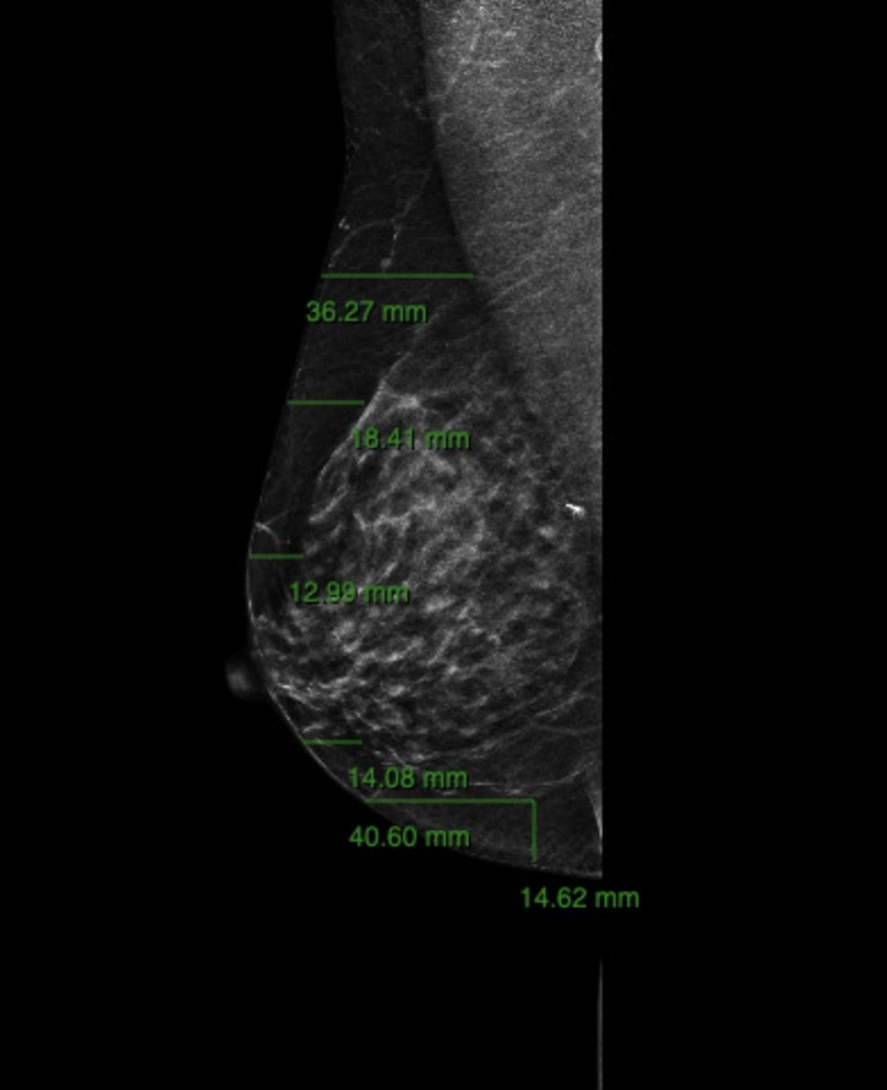
Additional radiological assessment of tissue thickness on the craniocaudal (CC) projection. Measurements are obtained at the level of the lateral radial incision site, corresponding to the junction of the outer breast quadrants, and integrated with those taken on the MLO projection at the inframammary fold. These preoperative radiological data are compared with intraoperative flap thickness to evaluate the correspondence between imaging-based and surgical measurements.

Preoperative imaging was performed within three months before surgery using standard digital mammography, ultrasound (US) and Magnetic Resonance Imaging (MRI). Breast tissue thickness was measured though digital mammography on mediolateral oblique (40°–50° inclination) and craniocaudal projections using Carestream Vue Motion software. Each measurement extended from the skin surface to the anterior lamella of the superficial fascial system (**Figures 1**, **2**). Preoperative breast tissue coverage (P-BTC) was expressed in millimeters and calculated as the mean of five standardized measurement points described by Rancati et al. in the mediolateral oblique view (31). Based on these data, patients were classified according to the Breast Tissue Coverage Classification (BTCC): type 1 (< 1 cm), type 2 (1–2 cm), and type 3 (> 2 cm) (**Table 1**). Additional measurements were obtained in the craniocaudal projection at the level of the radial incision site at the junction of the outer breast quadrants, and at the inframammary fold in the mediolateral oblique projection (31).

**Table 1:** Breast tissue coverage classification (BTCC) based on preoperative digital mammography.

Breast tissue coverage classification	N°
Type 1 (< 1 cm)	173
Type 2 (1–2 cm)	1337
Type 3 (> 2 cm)	153

The classification stratifies patients into three categories according to measured tissue thickness: Type 1 (<1 cm), Type 2 (1–2 cm), and Type 3 (>2 cm). These radiological categories are generally used to estimate preoperative breast tissue coverage and assist in surgical planning, particularly to evaluate suitability for prepectoral direct-to-implant reconstruction.

All mastectomies were performed by four senior breast surgeons. A single intraoperative dose of cefazolin (2 g) was administered for antibiotic prophylaxis. NSM incisions were mostly radial (92%), and the inframammary approach was the second choice for incisions. SRM procedures were performed using the inverted-T pattern, selected based on the degree of ptosis (Regnault classification (30)), macromastia, or patient preference for breast volume reduction. Conservative mastectomies were conducted along the anatomical dissection plane immediately beneath Camper’s fascia, or at the level of the breast capsule when the fascial layer was not identifiable.

Mastectomy flap thickness was assessed intraoperatively using a standardized method. After manual inspection to assess flap homogeneity, thickness was measured with a sterile ruler at the incision site by identifying the thinnest and the thickest points of the mastectomy flap. The final flap thickness value was calculated as the mean of these two measurements and used for group stratification. Implant selection was individualized based on specimen weight, breast footprint, pocket compliance, and contralateral breast symmetry. All patients underwent immediate prepectoral reconstruction using anatomically shaped polyurethane-coated implants (Microthane Sublime Line, Polytech, Dieburg, Germany).

Intraoperative perfusion assessment was routinely performed with indocyanine green (ICG) angiography to confirm flap vascularization. When perfusion was deemed adequate, the implant was placed in a prepectoral pocket even in cases with flaps thinner than 1 cm, following the protocol described by the authors in previous studies (24). If perfusion was insufficient, the procedure was converted to a two-stage reconstruction using a tissue expander; such cases were excluded from this analysis.

One or two 15-Fr J-VAC drains were positioned and maintained under suction. Before placement, the implants were irrigated with 10 cc of rifamycin solution (250 mg/3 ml), and the surgical field disinfected with 10% povidone-iodine (Betadine^®^, Mundipharma, Cambridge, UK). Gloves were routinely changed prior to implant insertion to minimize contamination risk.

### Postoperative management

The mean hospital stay was 2 days. A soft postoperative bra was applied on the first postoperative day to provide gentle compression and support. After discharge, patients were followed up to twice a week for the first three weeks to monitor early postoperative recovery and detect potential complications. Drain management followed a standardized protocol: drains were removed once the daily output was less than 20–25 mL to minimize the risk of seroma formation. A postoperative ultrasound examination was routinely performed a few days after drain removal to identify any residual serous collections.

Skin sutures were removed on postoperative day 14. Patients were advised to progressively resume normal daily activities after approximately two weeks and to return to full physical activity around one month postoperatively.

### Statistical analysis

Data were analyzed using IBM SPSS Statistics (version 31, IBM Corp., Armonk, NY, USA). Categorical variables were analyzed with chi-square or Fisher’s exact test. A *p*-value < 0.05 was considered statistically significant.

## Results

Patients had a mean age of 53 years (range 24–88 years) and a mean BMI of 24.07 kg/m² (range, 18.03–33.91). The mean mastectomy weight was about 300 grams (range 83–1040 grams), and the mean volume of breast implants was 320 cc (range 95–615 cc).

P-BTC and intraoperative flap thickness were evaluated for all 1,663 mastectomies. The mean intraoperative flap thickness was 10.1 mm (range 3–25 mm), while the mean radiological thickness according to the Rancati measurements (19) was 15.1 mm (range 6–28 mm). Based on intraoperative measurements, patients were stratified into three groups: Group 1 (5–6 mm, n = 143, 8.6%), Group 2 (7–9 mm, n = 585, 35.18%), and Group 3 (> 10 mm, n = 935, 56.22%). The distribution according to the Rancati classification (19) was R1: 10.4% (n = 173), R2: 80.4% (n = 1337), and R3: 9.2% (n = 153).

When comparing intraoperative and radiological classifications, no consistent correspondence was found. While Groups 1 and 2 together represented approximately 43% of mastectomies intraoperatively, only 10.4% were classified as R1 radiologically. This discrepancy indicates that preoperative imaging tends to overestimate flap thickness compared to intraoperative assessment. Several factors may contribute to this discrepancy. First, imaging quantifies the distance from the skin to the anterior lamella of the superficial fascia system, but the true surgical dissection plane may lie slightly more superficial, especially in patients with variable fascial integrity or in areas where the fascia is not clearly identifiable. Moreover, differences in projection angles, breast positioning, and the intrinsic resolution limits of mammography may also affect thickness estimation. Together, these elements can lead to a radiological classification that systematically assigns patients to higher Breast Tissue Coverage Classification (BTCC) categories than what is actually found in the operative field. Given these considerations, the lack of direct correlation between radiological and intraoperative classifications is not unexpected. Preoperative imaging remains valuable for overall surgical planning, but intraoperative evaluation continues to be the most accurate method for assessing the true thickness and perfusion quality of mastectomy flaps, which are critical determinants in reconstructive decision-making (**Table 2**).

**Table 2:** Distribution of mastectomies according to intraoperative flap thickness groups (Group 1: 5–6 mm; Group 2: 7–9 mm; Group 3: ≥10 mm) and corresponding radiological Breast Tissue Coverage Classification (BTCC) categories (R1–R3).

Intraoperative flap thickness (groups)	Rancati type 1 (<1 cm)	Rancati type 2 (1–2 cm)	Rancati type 3 (>2 cm)	Total mastectomies
Group 1 (5–6 mm)	36 (25.17%)	107 (74.83%)		143
Group 2 (7–9 mm)	97 (16.58%)	447 (76.41%)	41 (7.01%)	585
Group 3 (≥ 10 mm)	40 (4.27%)	783 (83.74%)	112 (11.99%)	935

The table illustrates the lack of direct concordance between radiological and intraoperative assessments, highlighting the tendency of preoperative imaging to overestimate flap thickness compared with real-time surgical evaluation.

Early postoperative complications were observed in 152 mastectomies (9.14%). When stratified by intraoperative flap thickness, no significant differences were found in the incidence of wound dehiscence (2.8% in Group 1, 2.05% in Group 2, 1.6% in Group 3; p = 0.566) or major site infections (0.7%, 1.54%, and 1.18%, respectively; p = 0.678). Implant extrusion was rare and did not differ among groups (p = 0.856).

Ischemic suffering of the mastectomy flap requiring surgical revision occurred more frequently in thinner flaps (2.1% in Group 1 vs. 0.51% and 0.43% in Groups 2 and 3). This trend approached statistical significance (p = 0.052) but remained low in absolute terms (< 3% in all subgroups).

Minor complications occurred in 88 cases (5.29% of mastectomies) and were evenly distributed across thickness groups (4.9%, 5.3%, and 5.35%; p = 0.975). When specifically analyzing periprosthetic seromas, the incidence was 0.7% in Group 1, 1.54% in Group 2, and 1.39% in Group 3; this difference reached statistical significance (p = 0.010).

Overall complication rates (major + minor) did not differ significantly among groups (10.48% in Group 1, 9.57% in Group 2, and 8.66% in Group 3; p = 0.704), indicating that intraoperative flap thickness is not independently associated with early postoperative morbidity following prepectoral DTI breast reconstruction (**Table 3**).

**Table 3:** Overall complication rates stratified by mastectomy flap thickness at the incision site.

	Group 1 (5–6 mm) (n = 143)	Group 2 (7–9 mm) (n = 585)	Group 3 (> 10 mm) (n = 935)	Total (n = 1663)	P-value
Wound dehiscence	2.8% (4)	2.05% (12)	1.6% (15)	1.86% (31)	0.566
Ischemic suffering of mastectomy flaps requiring revision	2.1% (3)	0.51% (3)	0.43% (4)	0.6% (10)	0.052
Major site infection	0.7% (1)	1.54% (9)	1.18% (11)	1.26% (21)	0.678
Implant extrusion	0	0.17% (1)	0.11% (1)	0.12% (2)	0.856
Minor complications	4.9% (7)	5.3% (31)	5.35% (50)	5.29% (88)	0.975
• Periprosthetic seromas	0.7% (1)	1.54% (9)	1.39% (13)	1.38% (23)	< 0.05
Any Complication	10.48% (15)	9.57% (56)	8.66% (81)	9.14% (152)	0.704

“Major site infection” refers to infections that required hospital admission for systemic antibiotic therapy and were therefore classified as major complications.

## Discussion

Prepectoral implant-based breast reconstruction (IBR) represents one of the most significant evolutions in modern oncoplastic surgery. Initially described by Snyderman and Guthrie in the early 1970s (32), the subcutaneous implant placement technique was abandoned because of high rates of capsular contracture and implant exposure. The subsequent rise of subpectoral reconstruction—providing muscular coverage and reduced implant visibility—dominated for decades. However, the drawbacks of muscle elevation, animation deformity, and chronic pain prompted a renewed interest in prepectoral approaches, now made possible by improved assessment of flap viability and many devices including acellular dermal matrices (ADMs), and polyurethane-coated implants (33).

The subpectoral approach, long considered the gold standard, ensures implant protection but at the cost of postoperative discomfort and muscle dysfunction. In contrast, the prepectoral plane preserves pectoralis major anatomy, decreases pain, and eliminates animation deformity (34, 35). Systematic reviews and meta-analyses now confirm comparable safety. Ostapenko et al. reported no significant differences in global complication rates between prepectoral and subpectoral reconstructions, while prepectoral placement showed significantly fewer capsular contractures and animation deformities (36). Similarly, King et al. demonstrated lower implant failure and reoperation rates for prepectoral reconstruction compared with subpectoral placement, reinforcing its long-term reliability (37).

Franceschini et al. compared traditional subpectoral IBR with prepectoral DTI reconstruction using polyurethane-coated implants. The prepectoral group demonstrated equal complication rates, shorter operative time, and superior outcomes in pain and aesthetic satisfaction (7).

Flap thickness has historically been viewed as a major determinant of postoperative safety. Larson et al. first defined the non–breast-bearing subcutaneous layer of approximately 1 cm as the optimal dissection plane (38), while Frey et al. later identified sub-8 mm flaps as high-risk for ischemia (39). The Rancati classification system subsequently formalized flap thickness (R1–R3) as a predictive variable for reconstructive planning (19, 31). Yet, recent data challenge the notion that thin flaps alone predict complications. Garutti et al. found that even thicker flaps (>17 mm) could exhibit higher ischemic rates due to impaired perfusion (40). Our findings similarly indicate that vascularity, rather than thickness, dictates early safety outcomes.

Perfusion imaging has revolutionized intraoperative decision-making. Phillips et al. validated laser-assisted indocyanine-green (ICG) angiography as a precise predictor of necrosis with 90% sensitivity (41). These tools now guide intraoperative flap assessment, allowing for safe expansion of prepectoral indications even in thin R1 patients.

The rise of ADMs, synthetic meshes, and advanced implant coatings has enhanced the stability and biocompatibility of prepectoral reconstructions. The iBAG multicenter study, the largest to date (over 1400 cases) has confirmed low capsular contracture (2.1%) and implant loss (6.5%) rates, establishing the safety of ADM-assisted prepectoral reconstruction in routine practice (9). Despite the advantages of using ADMs, their use is not without limitations. Several studies (42–44) have reported increased rates of seroma formation, infectious complications, and red breast syndrome in ADM-assisted reconstructions, suggesting a greater inflammatory response and a higher risk of early morbidity. Moreover, the financial burden associated with ADMs remains considerable and may restrict their routine use, particularly in publicly funded healthcare systems.

Simultaneously, new scoring systems such as the Prepectoral Breast Reconstruction Assessment Score proposed by Lo Torto et al. provide quantitative tools for preoperative risk stratification, integrating flap thickness and patient comorbidities to predict reconstructive success (3). These advances reflect an increasingly individualized approach to surgical planning.

The introduction of polyurethane-coated implants has further reduced capsular contracture rate. Castel et al. demonstrated in a 30-year follow-up that polyurethane coating inversely correlates with contracture severity (45). De Vita et al. reported excellent outcomes with polyurethane-coated implants in prepectoral breast reconstruction, even in patients with mastectomy flaps as thin as 0.8 cm; however, their series included only 34 patients and represented an early preliminary experience (15). Subsequent studies, including those by Sigalove et al. (14), Franceschini et al. (7), and the multicenter iBAG study (9), confirmed the overall safety and reliability of the prepectoral approach but generally excluded patients with very thin flaps (R1, <1 cm).

Beyond surgical feasibility, oncological safety has been confirmed; Scardina et al. demonstrated that prepectoral reconstruction yields local recurrence-free and overall survival rates equivalent to subpectoral techniques (8). Furthermore, prepectoral DTI with polyurethane-coated implants has been successfully extended to large and ptotic breasts, offering a ‘lifting’ effect that minimizes the need for skin-reducing mastectomies (46). To mitigate ischemic risks in traditionally excluded thin flaps, the adoption of closed-incision negative pressure wound therapy (ciNPWT) has shown a significant protective role, drastically reducing major complications like dehiscence and necrosis in patients with flaps measuring 0.5–0.9 cm (24).

While existing evidence has progressively broadened the indications for prepectoral reconstruction, most available studies remain limited by small sample sizes, heterogeneous inclusion criteria, and the frequent use of ADMs or synthetic meshes. Moreover, most published series systematically exclude patients with very thin mastectomy flaps, resulting in a persistent knowledge gap precisely in the subgroup considered to be the most challenging. In this context, the strengths of the present study lie both in its scale and in its methodological rigor. With 1,239 patients and 1,663 mastectomies, this represents the largest cohort to date focusing specifically on prepectoral reconstruction performed with PU-coated implants without ADM or mesh support, thus providing concrete evidence of outcomes in a population traditionally considered high-risk. Furthermore, unlike prior reports, often preliminary or limited to early experiences, this analysis includes a standardized surgical protocol, systematic perfusion assessment with ICG angiography, and comprehensive postoperative monitoring. Collectively, these elements allow not only a more robust evaluation of safety but also challenge the long-standing assumption that flap thickness alone should determine reconstructive eligibility.

On the other hand, this study presents some limitations that warrant consideration. First, its retrospective, single-center design may limit generalizability, as surgical expertise, perfusion assessment practices, and postoperative management may differ across institutions. Most importantly, the analysis is restricted to early postoperative outcomes within 30 days and was specifically designed to evaluate immediate surgical safety. Consequently, long-term outcomes—such as capsular contracture, rippling, implant loss, aesthetic stability, and the need for secondary revision procedures—were not assessed. These endpoints are essential to fully validate the long-term safety and durability of prepectoral direct-to-implant reconstruction, particularly in patients with thin mastectomy flaps. Therefore, our findings should be interpreted as evidence of short-term feasibility rather than definitive long-term validation.

Second, although radiologic and intraoperative thickness measurements were systematically collected, the intrinsic variability of imaging modalities may influence the accuracy of preoperative risk stratification. Finally, all reconstructions were performed using polyurethane-coated implants, which may not reflect outcomes associated with other implant surfaces. These limitations underline the need for prospective, multicenter studies with long-term follow-up to validate our findings and further refine the role of thin-flap prepectoral breast reconstruction within contemporary oncoplastic practice.

## Conclusions

This large single-center study shows that thin mastectomy flaps <1 cm are not, by themselves, a predictor of early complications in prepectoral DTI breast reconstruction. Even in high-risk patients and in the absence of acellular dermal matrices or meshes, overall morbidity remained low and comparable across thickness groups. Although slightly higher ischemic events were observed in the thinnest flaps, their absolute incidence was minimal, confirming that intraoperative assessment of vascularity plays a more decisive role than preoperative radiologic thickness.

These results challenge the traditional view that R1 patients are unsuitable for prepectoral reconstruction and support expanding indications when adequate perfusion is confirmed. With a standardized surgical protocol, careful perfusion-guided decision-making, and the advantages of polyurethane-coated implants, safe reconstruction can be achieved even in thin envelopes.

Further prospective studies are needed to validate these findings, but reduced flap thickness alone should no longer be considered an absolute contraindication to prepectoral DTI reconstruction.

## Data Availability

The raw data supporting the conclusions of this article will be made available by the authors, without undue reservation.
